# Down-regulation of DPP4 by TGFβ1/miR29a-3p inhibited proliferation and promoted migration of ovarian cancer cells

**DOI:** 10.1007/s12672-023-00815-y

**Published:** 2023-10-31

**Authors:** Chong Liu, Zhao-Wei Gao, Ying-Qi Liu, Lan Yang, Xia-Nan Wu, Ke Dong, Xiao-Ming Zhu

**Affiliations:** 1 Department of Clinical Diagnosis, Tangdu Hospital, Air Force Military Medical University, Xinsi Road, Xi’an, 710038 China; 2 School of Basic Medical Sciences, Air Force Medical University, No. 4 Company, Xi’an, China; 3 Department of Obstetrics and Gynecology, Hainan Branch of PLA General Hospital, Jianglin Road, Sanya, 572022 China

**Keywords:** DPP4, miR-29a-3p, Ovarian cancer, Proliferation, Migration

## Abstract

**Objective:**

To explore the DPP4 expression changes and functions in ovarian cancer (OV), as well as the regulation mechanism for DDP4.

**Methods:**

GEPIA2, GSE18520, GSE26712 and UALCAN were used to analyze differences in DPP4 expression between OV tumors and control tissues. Serum DPP4 levels were measured by ELISA. The prognostic values of DPP4 were evaluated using a Kaplan–Meier (KM) plotter. Small interfering RNA was used for DPP4 knockdown in OVCAR-3 and SKOV-3 cells. CCK-8 and scratch healing assays were used to determine the cells’ proliferation and migration abilities. Flow cytometry (FCM) was used to detect the cell cycle and apoptosis. A dual-luciferase assay was designed to confirm the regulatory effect of miR-29a-3p on DPP4.

**Results:**

The expressions of DPP4 mRNA and protein were decreased in OV tumor tissues. Serum DPP4 levels decreased in OV patients. KM plotter analysis showed correlation between high DPP4 expression and a poor prognosis in OV patients. By targeting knockdown of DPP4, we found that OVCAR-3 and SKOV-3 cells’ proliferation was inhibited, while cell’s migration ability was significantly promoted. FCM analysis showed that DPP4 knockdown induced a decrease in the S phase. Furthermore, DPP4 was shown to be downregulated by miR-29a-3p and TGFβ1 in OVCAR-3 cells, and miR-29a-3p expression was upregulated by TGFβ1. The effects of miR-29a-3p and TGFβ1 on OVCAR-3 cells’ biological behaviors were consistent with DPP4 knockdown.

**Conclusion:**

DPP4 was downregulated in OV patients. DPP4 knockdown significantly inhibited OVCAR-3 and SKOV-3 cell proliferation and promoted cell migration. DDP4 can be downregulated by TGFβ1 through the upregulation of miR-29a-3p in OV cells.

## Introduction

Ovarian cancer (OV) is a common malignant tumor of the female reproductive system. Globally, the incidence and mortality rates of OV were 3.4% and 4.7% respectively [1]. The 5-year survival rate has remained around 40% for the past 30 years [2]. The mechanism of OV occurrence and development remains unclear. Additionally, issues such as drug resistance, recurrence and metastasis remain problems for clinical OV therapy. Thus, there is an urgent need to identify novel molecules that are involved in OV development revealing the mechanism and improving the clinical therapeutic effects.

Dipeptidyl peptidase 4 (DPP4, also known as CD26) is an intrinsic type II transmembrane glycoprotein and a member of the serine protease family. DPP4 exists in both cell membrane-anchored and soluble forms, occurring in the serum [3]. DPP4 cleaves X-proline dipeptides from the N-terminus of polypeptides and has a diverse range of substrates, including incretins, chemokines, growth factors and neuropeptides [4]. DPP4 has been associated with multiple biological progresses [5–8]. Previous studies showed that DPP4 expression levels were significantly altered in tumors and might play a vital role in cancer development [9, 10]. In this study, we analyzed the expression changes and potential prognostic value of DPP4 in OV. Furthermore, we investigated the effect of DPP4 knockdown on the biological behavior OV cancer cells. Moreover, we explore potential mechanisms involved in DDP4 expression regulation. This study may present evidence for DPP4 involvement in OV progression.

## Materials and methods

### Differential expression analysis of DPP4

Based on TCGA and GTEx database, GEPIA2 (http://gepia2.cancer-pku.cn) bioinformatic tool was used to analyze the change of DPP4 mRNA expression in 426 OV tumor tissues compared with 88 control non-tumor tissues. Select Cutoff as |Log2FC|= 0.5. GSE18520 and GSE26712 datasets were used to verify the results from GEPIA2. UALCAN (http://ualcan.path.uab.edu/) was used to analyze the DDP4 protein expression in OV based on the CPTAC (Clinical Proteomic Tumor Analysis Consortium) database. The basic information of GSE18520 and GSE26712 were list in Table 1.

**Table 1:** Ovarian cancer survival analysis data set information

Dateset	Sample type	Quantity (tumor/control)	Platforms
GSE26712	late-stage high-grade ovarian cancer	185/10	GPL570
GSE18520	Papillary serous ovarian adenocarcinoma	53/10	GPL570

### Serum DPP4 levels detection by ELISA assay

Ethical approval was obtained from the Ethics Committee of Tangdu Hospital, Fourth Military Medical University. Informed consent was exempted from this study. Serum from 24 OV patients including16 with high-grade serous ovarian cancer (HGSOC, age range: 47–76 years) and 8 with other forms of OV (age range: 45–68 years). Additionally, serum from 23 healthy subjects (age range: 42–75 years) was collected. Peripheral blood was collected and centrifuged at 4000 rpm and 4 °C for 5 min to obtain serum. The serum samples were stored at − 80 °C. Serum DPP4 levels were detected using an ELISA kit according to the operation manual (R&D Systems, DY1180). In short, the capture antibody was coated in a 96-well plate and incubated overnight at room temperature. After blocking for 2 h, serum samples were added to the plate and incubated for 2 h. Then, streptavidin-conjugated detection antibody was added and incubated for 2 h. Then streptavidin-HRP antibody was added and incubated for 20 min. Finally, the TMB substrate was added, and the optical density (OD_450nm_) was measured. Serum DPP4 levels were calculated according to the standard curves.

### Kaplan–Meier (KM) plotter analysis

The KM plotter bioinformatic tool (http://www.kmplot.com/analysis/index.php?p=service) [11] was used to evaluate the potential prognostic value of DPP4 expression in OV patients. The best performing cutoff value was computed by using “auto select best cutoff” model. The source data was based on TCGA and GEO datasets (GSE18520, GSE26712).

### Cell culture

Human ovarian cancer cell lines, OVCAR-3 and SKOV-3, were purchased from Procell Life Science &Technology Co., Ltd (Wuhan, China). OVCAR-3 and SKOV-3 were used to investigate the effect of DPP4 on the cells’ biological behavior. OVCAR-3 was cultured with RPMI-1640 (Gibco, Carlsbad, NY, USA) supplemented with 20% Fetal Bovine Serum fetal bovine serum (ExCell Bio, Shanghai, China). SKOV-3 was cultured with Dulbecco’s Modified Eagle Medium (Gibco) plus 10% Fetal Bovine Serum (ExCell Bio). The cells were cultured in a 5% CO_2_ incubator at 37 °C.

### Small-interfering RNA (siRNA) and miR-29a-3p transfection

siRNA (Sangon Biotech, Shanghai, China) was used to knock down the expression of DPP4. miR-29a-3p (Sangon Biotech) was used to investigate the effect of miR-29a-3p on DPP4 expression. Approximately 1 × 10^5^ cells were seeded in 6 well plates and cultured for,

24 h Subsequently siRNA was transfected into OVCAR-3 and SKOV3 cells, and miR-29a-3p mimics were transfected into OVCAR-3 cells. The siRNA and miR-29a-3p transfection were carried out using Lipofectamine 2000 (Invitrogen, Carlsbad, CA, USA) according to the manufacturer's instructions. The sequences are listed in Table 2.

**Table 2:** siRNA and miRNA sequences used in the study

	Sense (5'-3')	Antisense (5'-3')
siNC	UUC UCC GAA CGU GUC ACG UTT	ACG UCG CAC GUU CGG AGA ATT
siDPP4	CCAAGAAAUAUCCUCUACUAUTT	AUAGUAGAGGAUAUUUCUUGGTT
miRNC	UUGUACUACACAAAAGUACUG	GUACUUUUGUGUAGUACAAUU
miR29a-3p	UAGCACCAUCUGAAAUCGGUUA	ACCGAUUUCAGAUGGUGCUAUU

### Quantitative RT-PCR and western blot

After 48 h of siRNA or miR-29a-3p mimic transfection, RNA and protein were extracted from the cells. Total RNA was extracted from cells using a TRIzol Reagent Kit (GLPBIO, Cat: GK20008) For mRNA, cDNA was prepared using the Evo M-MLV Mix Kit (Accurate Biology AG) and cDNA for micro-RNA was prepared by using the Mir-X™ miRNA First-strand Synthesis Kit (Takara Bio, Japan). By using the Blastaq™ MasterMix kit (ABM, Canada), the reference genes for mRNA and miRNA were GAPDH and U6, respectively Quantitative real-time RT-PCR was used to detect the DPP4 mRNA and miR-29a-3p expression levels. Real-time PCR was performed on a QIAGEN Rotor-gene Q Real-Time PCR system (QIAGEN, Germany). Relative expression was calculated using the ΔΔCt method. U6 primers and miR29a-3p reverse primers were provided by the Mir-X™ miRNA First-Strand Synthesis Kit mentioned above. The primers for RT-PCR were list in Table 3.

**Table 3:** Prime sequences used for RT-PCR

Gene	Forward primer (5'-3')	Reverse primer (5'-3')
DPP4	TGGGCAACACAAGAAAGAA	TGTCTGTAACCTTCTTCAT
GAPDH	GGTGGTCTCCTCTGACTTCAACAG	GTTGCTGTAGCCAAATTCGTTGT
miR29a-3p	TAGCACCATCTGAAATCGGTTA	

For Western blotting, cell proteins were separated using SDS-PAGE and transferred onto a nitrocellulose membrane. The membranes were blocked for 2–3 h at room temperature, and then incubated overnight at 4 °C with an anti-DPP4 antibody (Cell Signaling Technology, Danvers, MA, USA). After washing with TBST three times, the membranes were incubated with an HRP labeled secondary antibody (ZSGB-BIO, Beijing, China) for 2 h. Proteins were detected using a Western-blotting luminol reagent (4A Biotech Co., Ltd, Beijing, China), GAPDH was used as the internal standard. Protein bands were visualized using a chemiluminescence detection system (Bio-Rad). The relative density of specific protein expression was determined using Image Lab software.

### CCK8 assay

The CCK8 assay was performed to detect cell proliferation according to the manufacturer's instructions (GLPBIO, Cat: GSK10001). First, 2 × 10^3^ cells were seeded in a 96-well plate. second, at different time points during culture (0, 24, 48, 72 h), 10μL of CCK8 reagent was added into each well and the cells were incubated for 2 h at 37 °C. Then, the OD_450nm_ was measured, and the relative cell viability was calculated. as a percentage using the formula: (mean OD450 of treated cells/mean OD450 of control cells) × 100%.

### Flow cytometry (FCM) analysis

Cells were collected 48 h after siRNA transfection for cell cycle and apoptosis detection. FCM was used to examine the cell cycle and apoptosis. For cell cycle analysis, cells were treated by using a cell cycle detection kit (KeyGENBiotech, Nanjing, China) according to the manufacturer's instructions. In brief, cells were fixed for 2 h with ethanol and stained with propidium iodide (PI) in buffer containing 10 μg RNase A. The cells were then kept in the dark at room temperature for 30 min. Cell cycle distribution was assessed using a FACS Calibur flow cytometer (BD Biosciences, San Jose, USA). Cell apoptosis was detected using the annexin V–FITC apoptosis detection kit (4A Biotech Co, Ltd, Beijing, China, Cat: FXP022). Cells were collected, washed twice with PBS and stained with annexin V-FITC and PI in the dark at room temperature for 15 min. The percentage of apoptotic cells was calculated using CellQuest 6.0 (BD Biosciences).

### Cell migration assay

As previously described [12], a scratch assay was used to investigate the cell’s migration ability. In brief, cells were seeded and cultured in a 6-well plate. The scratch was performed when the cell density reached 80–90%. A monolayer of cells was then scratched with a new 200μL pipette tip across the center of the well, and washed gently twice with PBS, Then, cells were cultured for 24 h without serum. The scratch images were captured using a Cytation1 (BioTek, Vermont, USA). The cell migration ratio = (start distant—end distant)/start distant.

### Luciferase reporter assay

To predict the binding sites between DPP4 and miR-29a-3p, TargetScan Human (https://www.targetscan.org/vert_71/) was used. The pGL3-DPP4-wild-type (WT) and pGL3-DPP4-mutant (MUT) were synthesized by Tsingke Biotechnology Co., Ltd (Beijing, China). Then, HEK293 cells were seeded into a 24-well plate and transiently co-transfected with synthesized plasmids (WT [1 µg] or MUT[1 µg]), pRL-TK-Renilla(20 ng), miR-29a-3p mimics(20 pM) or mimics-NC(20 pM) by using Lipofectamine™ 2000 (Invitrogen). After 48 h, the luciferase activities in cells were detected by using the Dual-Luciferase Reporter Assay Kit (Cat No. E1910, Promega, USA) and the Glomax luminescence detector (Promega). The results were expressed as the relative firefly luciferase activity, which is obtained after normalization to Renilla luciferase activity.

### Statistical analysis

Serum DPP4 levels were expressed as the mean ± standard error. Student’s t-test was used to analyze the difference of serum DPP4 levels between OV patients and controls. The data from cell experiments are expressed as the mean ± standard error. Student’s t-test was used to analyze the difference of the cell proliferation rate, cell migration, cell cycle and apoptosis between different groups. All experiments were confirmed in three biological replicates. p < 0.05 was considered to be statistically significant. Statistical analysis was performed using GraphPad Prism 5.01.

## Results

### DPP4 expression was decreased in OV patients

**Fig. 1:**
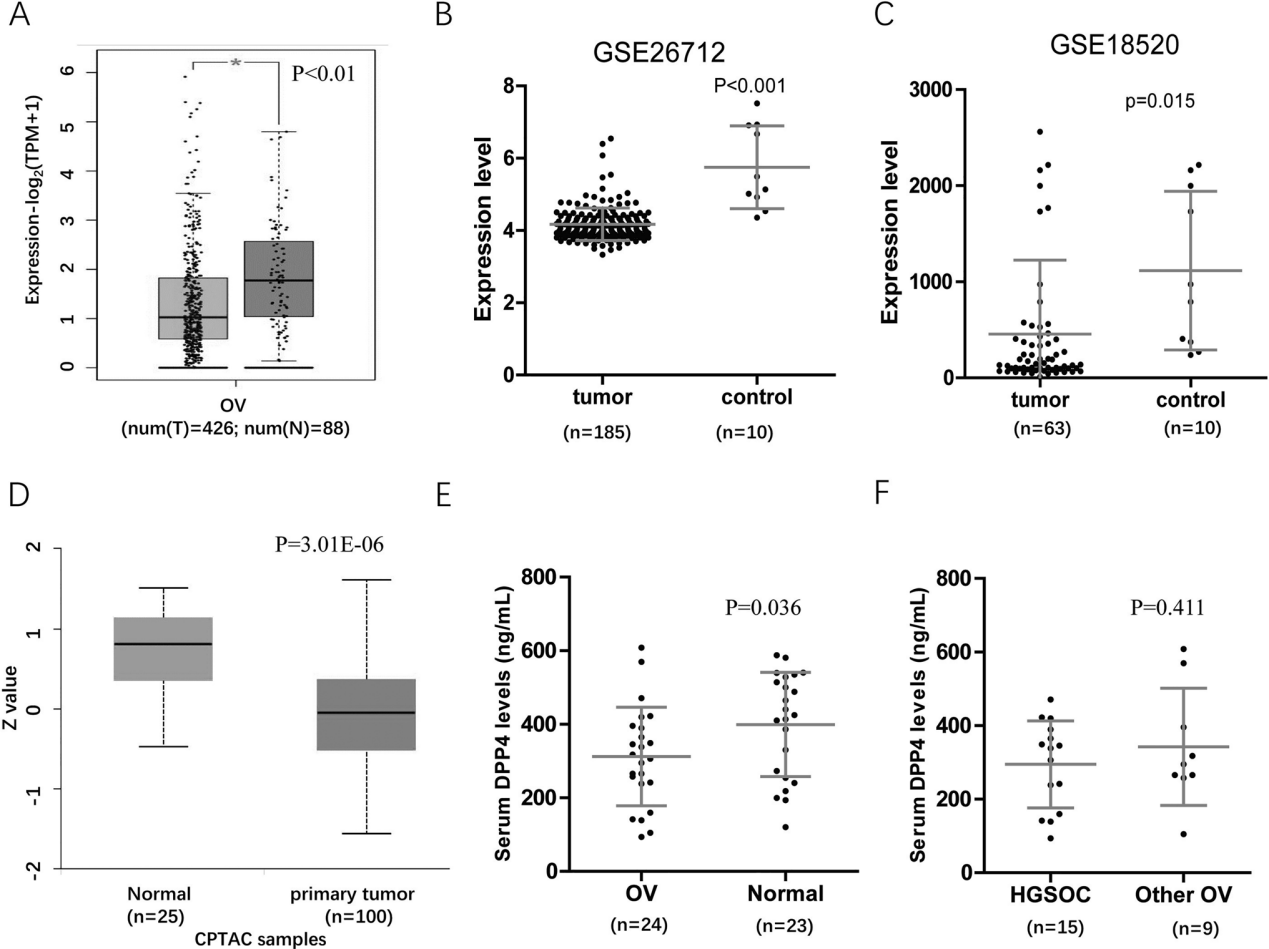
Expression difference analysis of DPP4 between OV and controls. **A** DPP4 mRNA expression was reduced in OV tumor tissues. *TPM* Transcripts per kilobase of exon model per million mapped reads. **B**, **C** Differential DPP4 expression between OV tumor tissues and controls in the GSE26712 and GSE18520 datasets. **D** The difference in DPP4 protein expression between OV tumor tissues and controls is based on the CPTAC database. **E** The serum DPP4 levels in OV patients. **F** The serum DPP4 levels in HGSOC and the other subtypes of OV

Based on the TCGA and GTEx databases, GEPIA2 analysis showed a decrease in DPP4 mRNA expression in OV tumor tissues (Fig. 1A). The results from the GSE26712 and GSE18520 datasets also showed downregulation of DPP4 expression in OV tumor tissues (Fig. 1B, C). Furthermore, based on the CPTAC database, UCLUC analysis showed a decrease in DPP4 protein expression in OV tumor tissues (Fig. 1D). We further investigated the DPP4 levels in the serum of OV patients. As shown in Fig. 1E, serum DPP4 levels were significantly lower in HGSOC patients than in healthy controls (HCs) (OV: 295 ± 118 ng/ml; HC: 399 ± 141 ng/mL, *p* = 0.023). There was no significant difference in serum DPP4 levels between HGSOC and other subtypes of OV (Fig. 1F).

### Prognostic value of DPP4 in OV patients

**Fig. 2:**
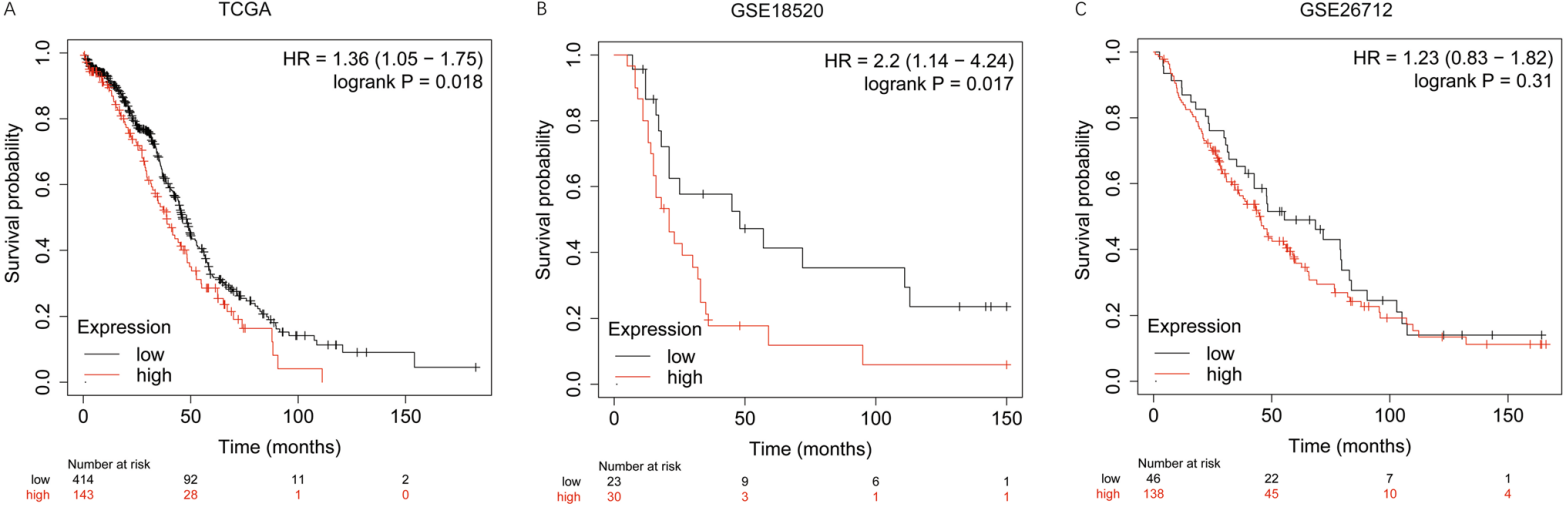
Prognostic value of DPP4 in OV patients based on different datasets. **A** Survival curves of DPP4 in TCGA. **B**, **C** Survival curves of DPP4 in the GSE18520 and GSE26712 datasets

The KM plotter was used to evaluate the relationship between DPP4 expression and prognosis. Based on the TCGA database (Fig. 2A), high DPP4 expression was correlated with poor overall survival (OS) of OV patients (hazards ratio [HR] 1.36; logrank p = 0.018). Based on the GEO database, the GSE18520 datasets showed a significant correlation between high DPP4 expression and poor OS (Fig. 2B), while in the GSE26712 datasets, there was no significant correlation between DPP4 expression and OS (Fig. 2C).

### DPP4 knockdown inhibited proliferation and promoted migration of OV cancer cells

**Fig. 3:**
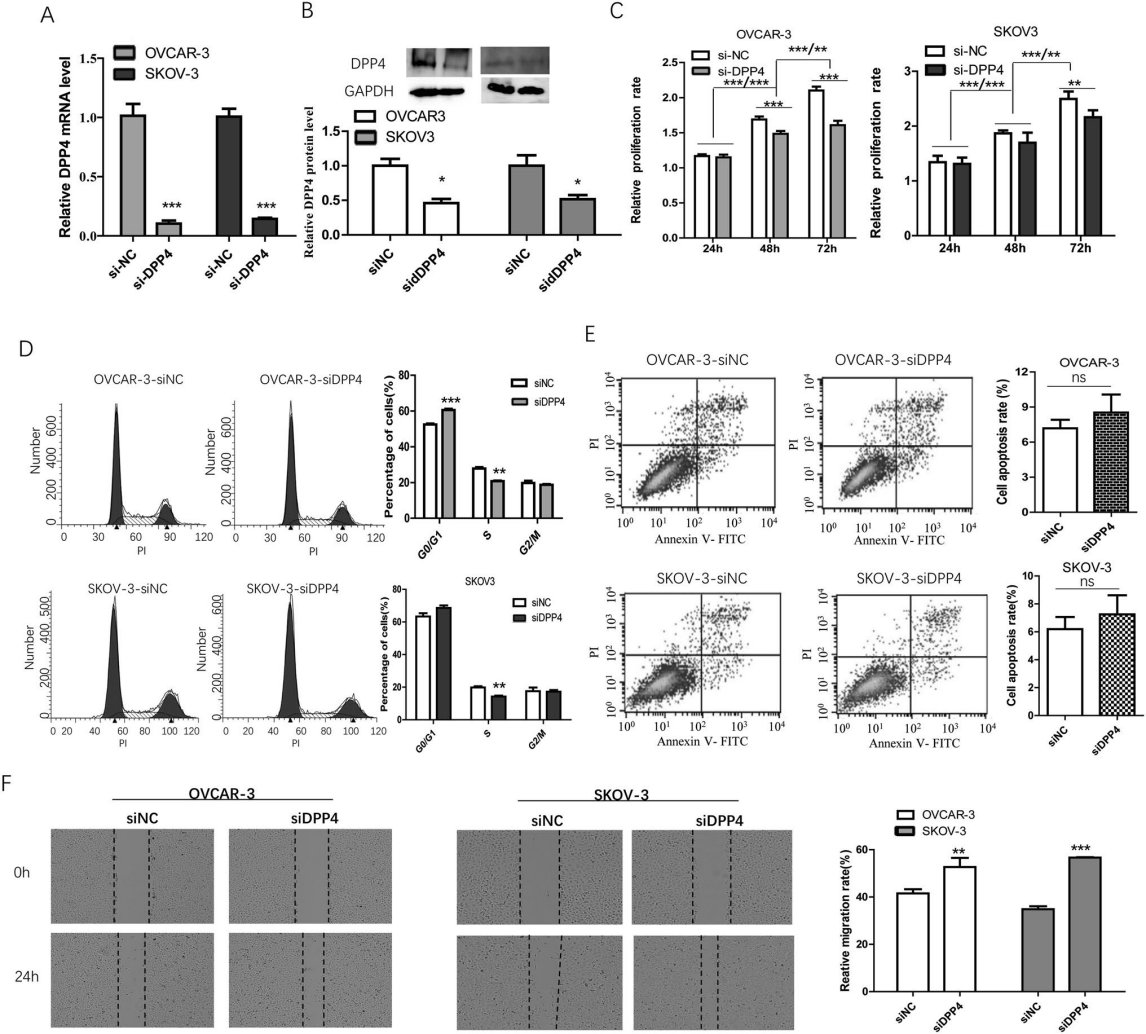
The effect of DPP4 knockdown on cell biological behaviors. **A**, **B** The inhibitory effect of siDPP4 transfection on DPP4 mRNA and protein expression levels. **C** DPP4 knockdown inhibited cell proliferation. **D** DPP4 knockdown resulted in a decrease of the cells in the S phase. **E** There was no significant effect of DPP4 knockdown on cell apoptosis. **F** DPP4 knockdown promoted cell migration

DPP4 mRNA and protein expression were significantly downregulated by siRNA transfection (Fig. 3A, B). By using the CCK8 assay, we found that OVCAR-3 and SKOV-3 cell proliferation was significantly inhibited by DPP4 knockdown (Fig. 3C). FCM analysis showed that there was a significant decrease in DPP4 knockdown cells in the S phase (Fig. 3D). There was no significant effect of DPP4 knockdown on OVCAR-3 and SKOV-3 cell apoptosis (Fig. 3E). Furthermore, a scratch assay was performed to investigate the effect of DPP4 knockdown on the migration of OVCAR-3 and SKOV-3 cells. As show in Fig. 3F, DPP4 knockdown significantly promoted the cell’s migration ability.

### DPP4 was downregulated by TGFβ1/miR-29a-3p

**Fig. 4:**
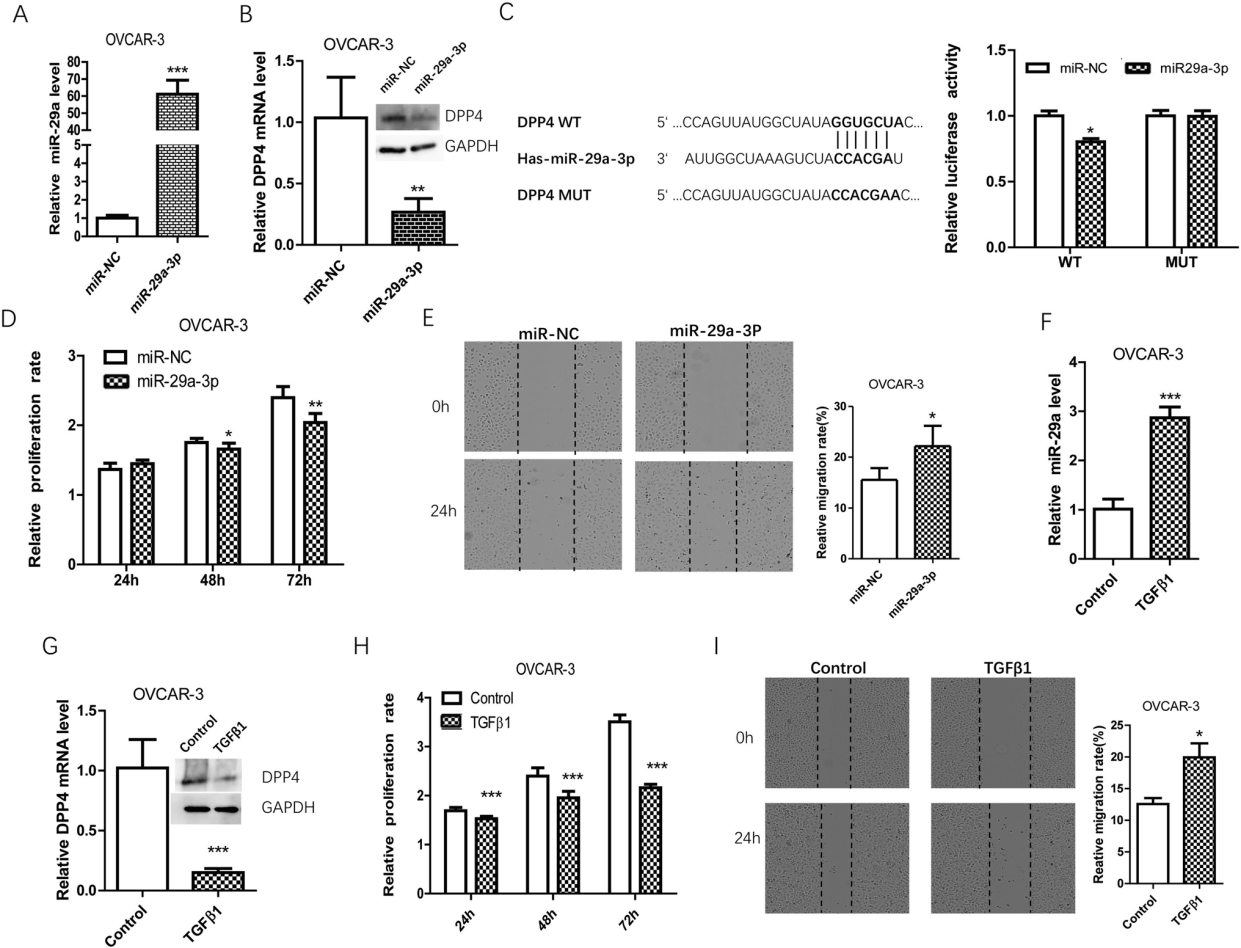
The effect and mechanism of TGFβ1 on DPP4 expression and on cell biologic behaviors. **A**, **B** The effect of miR-29a-3p on DPP4 mRNA and protein expression. **C** Dual-luciferase assay verified the target binding of miR-29a-3p and DPP4. **D** miR-29a-3p inhibited cell proliferation. **E** miR-29a-3p promotes cell migration. **F** miR-29a-3p expression was upregulated by TGFβ1 treatment. **G** TGFβ1 treatment inhibited DPP4 expression. **H** TGFβ1 treatment inhibited cell proliferation. **I** TGFβ1 treatment promoted cell migration

To explore the potential mechanism behind DPP4 downregulated in OV, TargetScan was employed to predict the regulators of DPP4 and miR-29a-3p was predicted to be a potential regulator of DPP4. In OVCAR-3 cells, DDP4 expression was significantly inhibited by miR-29a-3p mimics transfection (Fig. 4A, B). Then, adual-luciferase assay was designed to confirm the regulatory effect of miR-29a-3p on DPP4. The cells cotransfected with miR-29a-3p mimics and DPP4-WT showed significantly lower luciferase activity than those cotransfected with mimics-NC and DPP4-WT (*p* < 0.01), while cells transfected with DPP4-MUT exhibited no obvious changes in luciferase activity (Fig. 4C). Furthermore, miR-29a-3p mimics inhibited OVCAR-3 cell proliferation and promoted cell migration, which was consistent effects with si-DPP4 (Fig. 4D, E). Notably, miR-29a-3p is known to be a downstream target of TGF-β1[13]. Thus, we investigated the changes in miR-29a-3p and DPP4 expression in response to TGFβ1 stimulation. We found that TGFβ1 treatment significantly promoted miR-29a-3p expression and inhibited DPP4 expression (Fig. 4F, G). Furthermore, TGFβ1 treatment inhibited OVCAR-3 cell proliferation and promoted cell migration (Fig. 4H, I). Taken together, these results indicated that the TGFβ1/miR-29a-3p might be involved in DPP4 downregulation and OV development.

## Discussion

Ovarian cancer is one of the major causes of cancer-related deaths among women worldwide. Targeted therapies have shown a good clinical effect for certain OV patients, such as PARP inhibitors [14]. However, due to the complexity and heterogeneity of tumors, there is still an urgent need to find new therapeutic targets for OV. In this study, we analyzed the changes in DPP4 levels in OV patients and the effect of DPP4 on OVCAR-3 and SKOV-3 cells’ behavior. The major findings are summarized as follows: First, DPP4 levels were reduced in OV patients Second, DPP4 knockdown significantly affected the proliferation and migration of OVCAR-3 and SKOV-3 cells. Third, the TGFβ1-DPP4 might play an important role in OV development.

Based on the TCGA, GEO and CPTAC databases, we found a significant decrease in DPP4 expression in OV tumor tissues compared to control tissues. Additionally, serum DPP4 levels were significantly lower in OV patients than in healthy subjects. Previous studies have reported a decrease in serum DDP4 levels in several cancer types, such as gastric cancer, and colorectal cancer [3, 15]. Boccardi and colleagues showed that serum DPP4 levels were decreased in gastric cancer and may serve as an early detection marker [3]. Elzefzafy et al.’s study showed that serum DPP4 levels were lower in colorectal cancer compared to colorectal diseases and healthy controls [15]. Notably, Shao et al.’s study showed that serum CD26 levels were decreased in high-grade serous ovarian carcinoma patients, and may serve as an independent diagnostic marker [16]. For prognosis, based on the TCGA database, OV patients with high DPP4 mRNA expression levels showed a poor prognosis. Base on the GEO database, high DPP4 expression in the GSE18520 dataset was associated with a poor prognosis of ovarian cancer, while DPP4 expression in the GSE26712 was not associated with a prognosis. Taken together, these data demonstrated that DPP4 levels were decreased in OV patients (both in tumor tissues and serum). However, the prognostic value of DPP4 requires further verification, as there may be more confounding factors influencing the prognosis of OV patients, including CD26 expression.

To explore the roles of DPP4 in OV, we further investigated the effects of DPP4 knockdown on OVCAR3 and SKOV-3 cells’ proliferation and migration. By using si-DPP4 transfection, we observed that DPP4 knockdown significantly inhibited cell’s proliferation, which might be due to a decrease in cell numbers in the S phase. Consistently, previous studies have shown the inhibitory effects of DPP4 knockdown on the proliferation of other cancer cells [9, 10, 17]. For example, Okamoto T and colleagues found that DPP4 knockdown significantly inhibited the proliferation of malignant pleural mesothelioma [17]. Hu X’s study showed an inhibitory effect of DPP4 knockdown on papillary thyroid carcinoma cell’s proliferation [9].Furthermore, Yang et al.’s study showed that DPP4 overexpression promotes endometrial carcinoma cell proliferation [18]. These results suggest that DPP4 overexpression may promote OV cell proliferation, which could contribute to the poor prognosis in OV patients.

The effect of DPP4 on cell migration appears different in different cancer types. Our results in this study and Yang’s report [19] showed the promoted effect of DPP4 knockdown on OV cell lines and 4 T-1 (breast cancer cell line) cell migration respectively. However, Ng et al. found that DPP4 knockdown significantly inhibited the migration of colorectal carcinoma cells (HT29) [20]. Epithelial-mesenchymal transition (EMT) plays an important role in facilitating cancer cell migration. Yang et al. found that DPP4 knockdown induces EMT in breast cancer cells [19]. However, Hu X’s study showed the inhibited effect of DPP4 knockdown on the EMT of papillary thyroid carcinoma cells [9]. Taken together, there were significant differences in the effect of DPP4 on the migration and EMT progress among different types of cancer cells, which demonstrated CD26’s complex roles involved in cancer development. The correlation between DPP4 and EMT in OV cancer needs further study.

The mechanism of DPP4 expression regulation remains unclear. Chen Z’s study has shown the negative regulation of DPP4 expression by TGFβ1 in keloid-derived fibroblasts [21]. In the present study, we found that TGFβ1 treatment reduced DPP4 expression in OVCAR-3 cells. Furthermore, the effects of TGFβ1 on OVCAR-3 cell behavior were consistent with the outcomes observed DDP4 knockdown by siRNA. These data indicated the important role of TGFβ1-DPP4 signaling in OV development. Notably, miR-29a-3p might serve as an intermediary molecule between TGFβ1 and DPP4. In OVCAR-3 cells, TGFβ1 upregulated miR-29a-3p and miR-29a-3p inhibited DPP4 expression. The regulatory effect of miR-29a-3p on DPP4 was confirmed by the dual-luciferase assay in this study. Thus, these results indicated that DPP4 is downregulated by TGFβ1/miR-29a-3p in OV cells. Furthermore, the effects of miR-29a-3p and TGFβ1 on OVCAR-3 cells’ biological behaviors were consistent with DPP4 knockdown using si-DPP4. In contrast to this study, previous studies have shown downregulation of miR-29a-3p in response to TGFβ1 in human fetal lung fibroblast IMR-90 cells and hepatic stellate cell line LX-2 [13, 22]. Thus, the relationship between TGFβ1 and miR-29a-3p exhibits variability variation in different cell types.

There are some limitations to this study. First, the function of CD26 on OV progression was not evaluated in animal models. Thus, the efficacy of targeting CD26 therapy is also unknown for OV. Second, the effects of siDPP4 on downstream molecules were also unclear in this study, and these data could be further studied by using omics screening.

In conclusion, our study showed reduced levels of DPP4 levels in OV. Furthermore, DPP4 knockdown could inhibit OVCAR-3 cell’s proliferation and promote cell’s migration. DDP4 could be downregulated by TGFβ1 through the upregulation of miR-29a-3p in OV cells. Taken together, our study presents the potential roles of DPP4 in OV development.

## Data Availability

All data generated or analysed during this study are included in this published article.
